# The Relationship between Physical Activity, Self-Perceived Health, and Cognitive Function in Older Adults

**DOI:** 10.3390/brainsci11040492

**Published:** 2021-04-13

**Authors:** Radka Dostálová, Chelsea Stillman, Kirk I. Erickson, Pavel Slepička, Jiří Mudrák

**Affiliations:** 1 Faculty of Physical Culture, Palacký University Olomouc, 771 11 Olomuniec, Czech Republic; 2 Department of Psychology, University of Pittsburgh, Pittsburgh, PA 15260, USA; cms289@pitt.edu (C.S.); kiericks@pitt.edu (K.I.E.); 3 College of Science, Health, Engineering, and Education, Murdoch University, Murdoch, WA 6150, Australia; 4 Department of Pedagogy, Psychology and Didactics, Faculty of Physical Education and Sport, Charles University, 162 52 Prague, Czech Republic; Slepicka@ftvs.cuni.cz (P.S.); mudrak@ftvs.cuni.cz (J.M.)

**Keywords:** aging, cognitive function, processing speed, physical activity, self-perceived health, Vienna test system

## Abstract

There are consistent associations between physical activity and self-perceived health. However, it is not clear whether associations between self-perceived health and participation in physical activity could be accounted for by associations with cognitive function. In the present study, we examined whether associations between physical activity and cognitive functioning could explain the variability between physical activity and self-perceived health. A sample of 204 older adults performed three cognitive tests selected from the Vienna test system battery: The Determination, Cognitrone, and Visual Memory tests. These tests measure general processing speed, attention, and visual memory, respectively. Participants also completed the 12-item Short Form Health Questionnaire SF-12 to measure perceived health, and the Physical Activity Survey for the Elderly to measure physical activity. Linear regressions and the PROCESS macro for SPSS were used to test our hypotheses. Consistent with our hypotheses, processing speed accounted for significant variance in the relationship between physical activity and self-perceived health. This suggests that cognitive processing speed might be an indirect path by which physical activity relates to enhanced health perceptions. The results demonstrate that associations between physical activity and self-perceptions of health are related to a fundamental cognitive process.

## 1. Introduction

As the average age of the world population rises, the topic of healthy aging receives ever-growing research attention [1,2,3,4,5]. Active and healthy aging is a multi-factorial process that has become a health policy priority for local, national, and international health authorities in order to reduce the growing incidence of dependence. Physical activity should be included in lifestyles of people of all ages, because it represents one of the most important factors of active and healthy aging [5,6,7].

Greater amounts of physical activity are associated with higher ratings of perceived health [8]. Self-perceived health is a complex construct that refers to the way in which numerous aspects of health, both subjective and objective, are combined within the perceptual framework of the individual respondent [9]. Mudrák et al. [10] describe perceived health as a state of how one feels, and how one experiences and evaluates their state of health. Notably, this perception may not always be in line with objectively determined states of heath. For this reason, the process of assessing self-perceived health is complex and multifaceted, unlike objective (e.g., medical) assessments that are primarily focused on physical health. Fortunately, questionnaires such as the 12-item Short Form Health Questionnaire SF-12 [11] have been designed to measure both the physical and mental/emotional aspects of self-perceived health, as well as related concepts such as the health-related quality of life.

The topic of self-perceived health is becoming increasingly popular in both biomedical and psychosocial research fields, because subjective health ratings have been shown to correlate with and even precede objective health outcomes [3,9]. For example, Rodin and McAway [12] identified self-perceived health as an important predictor of life expectancy. In addition, changes in self-perceived health in the cognitive domain, also known as subjective cognitive decline, have been associated with higher risk of dementia, even in clinically normal individuals [13,14,15]. This evidence suggests that a person’s insight into their own overall health may have important implications for forecasting their future health needs [13]. Indeed, self-perceptions and awareness of one’s own functioning might be highly dependent on cognitive abilities.

One important factor linked to both subjective and objective health—across both cognitive and physical domains—is physical activity. Physical activity significantly reduces the risk of heart disease, stroke, high blood pressure, osteoporosis, and various types of cancers, among many other health benefits [7]. Physical activity is also linked to improved cognitive functioning and brain health across the lifespan [16]. For example, cross-sectional and experimental studies [17,18,19,20] show that greater amounts of physical activity are related to better cognitive functioning in older adults. The relationship between more physical activity and better cognitive performance is thought to be mediated by improved brain integrity, including increased cerebral blood flow [21,22], volume [23], and white matter integrity [24]. The salutary effects of physical activity on cognition are particularly pronounced in the domains of general processing speed, memory, and executive functioning [25,26]. Attention, processing speed, and memory are cognitive functions that are related to the self-sufficiency of older people. These cognitive domains might also be the most impacted by age [27,28]. Given the wide-reaching effects of physical activity on physical and cognitive (via brain) health, it is perhaps unsurprising that higher levels of physical activity have been shown to correlate with higher self-perceived health. For example, Mudrák et al. [3] showed that higher moderate-to-vigorous physical activity levels amongst 212 Czech seniors were associated with higher levels of self-perceived health, while Kaleta et al. [29] demonstrated a similar relationship between self-perceived health and leisure-time physical activity.

Despite the consistent associations observed between physical activity and self-perceived health, the mechanisms linking physical activity and self-perceived health are still unclear. Furthermore, it is not clear from this existing work whether changes in self-perceived health associated with participation in physical activity interventions could be accounted for by changes in central (i.e., cognitive) processing. Therefore, a central goal of the present study was to assess whether the relationship between physical activity and self-perceived health can be accounted for by variability in cognitive function. Based on the prior physical activity intervention work, in this cross-sectional study we hypothesized that associations between greater amounts of physical activity and perceptions of health would be statistically accounted for by variability in cognitive functioning (e.g., processing speed).

## 2. Method

### 2.1. Sample

The research sample consisted of 204 older adults aged between 60 and 89 years (mean = 70.02, SD = 6.092). Participants were recruited via email through various physical activity programs for older adults in which they actively participated, and senior’s clubs without a focus on physical activity. All participants were retired; on average, they had spent 12.2 years in retirement. The majority of respondents were women (75%), which may reflect a greater interest of women in physical activity or maintaining social contacts during visits to the senior’s clubs. A greater number of female participants (75%) is a common phenomenon in health-related research, which could be related to both higher rates of volunteering and health consciousness in females. Over half (55.9%) of the participants had a high-school education, and 22.5% had a university education. The sample size was selected based on prior studies of physical activity and cognition and perceived health [3,20].

Inclusion criteria included: participants being (1) 60+ years old; (2) ambulatory; and cognitively normal. Exclusion criteria included: (1) participants under 60 years old; (2) having a physical disability that prevented physical activity; and (3) diagnosis of a cognitive impairment or disability.

Current physical activity levels were not exclusion criteria; therefore, the research sample consisted of a wide range of physical activity levels. The demographic characteristics of the sample are presented in Table 1.

### 2.2. Methods

Researchers traveled to seniors’ clubs in and around Prague and administered paper and pencil questionnaires and computerized cognitive tasks to study participants. The specific tasks and questionnaires administered are further described below. The testing sessions lasted approximately 45 min. These sessions occurred in a quiet and private testing space. Participants were compensated for their time with a small gift (a pencil with the university logo) but were otherwise unpaid volunteers. Informed consent was obtained in accordance with the principles outlined in the Declaration of Helsinki. The Institutional Review Board at the Charles University in Prague approved all study procedures (IRB approval number is 225/2016).

#### 2.2.1. Vienna Test System

The Vienna test is a comprehensive battery of electronically administered achievement tests on a laptop which measure a wide range of cognitive functions, such as attention, working memory capacity, reaction speed, spatial abilities, and complex cognitive function and personality characteristics. The participants performed three cognitive tests selected from the Vienna test system battery: determination test (DT), Cognitrone (COG) and visual memory test (VISGED). The selection of these tests from the Vienna test system was based upon the cognitive domains showing the largest changes during aging. The reliability of all tests is very high (*r* = 0.65–0.99), and validity has been verified by a number of studies [30].

**Figure 1:**
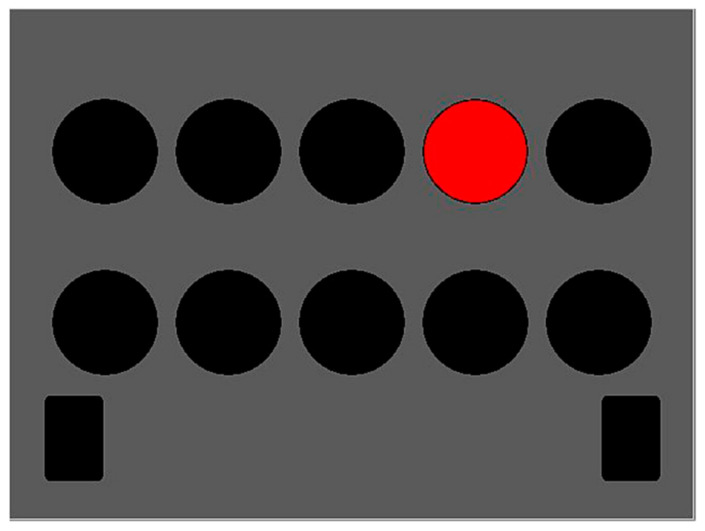
Determination test.

The determination test (Figure 1) is a complex instrument measuring basic processing speed by demanding fast and accurate responses to changing visual and acoustic stimuli, which are presented randomly. The participants are instructed to react as quickly as possible to visual or auditory stimuli by pressing corresponding buttons on a response panel. There are five visual stimuli colored white, yellow, red, green, and blue, which appear in an upper and a lower row. The buttons assigned to these five colors are arranged on the response panel in such a way that the respondent can use both hands. There are two additional visual stimuli, in the form of white, rectangular, visually distinct fields that appear in the bottom left- and right-hand corners of the screen, to which the respondent must react by pressing the corresponding (left or right) foot pedal. Two acoustic stimuli (high and low tone) are assigned to two “sound” buttons in the middle of the panel. The lower rectangular black button is pressed for a low tone, and the upper rectangular grey button for a high tone. In the context of a single pairing of stimulus and response, the skills required are not particularly difficult. The difficulty of the DT arises from the need to sustain continuous, rapid, and varying responses to rapidly changing stimuli. The speed of the presentation of the stimulus is automatically adapted to the level of respondent’s performance so that the subjective difficulty of the test was always high [31]. Due to its structure, the test is especially suitable for examining cognitive changes taking place in older adults because the ability to solve these tasks is vulnerable to the process of aging [32,33,34]. The main outcome from the DT is performance accuracy [4]. A higher score indicates better processing speed. The reliability of the test is very high, ranging from *r* = 0.98 to *r* = 0.99 [30].

**Figure 2:**
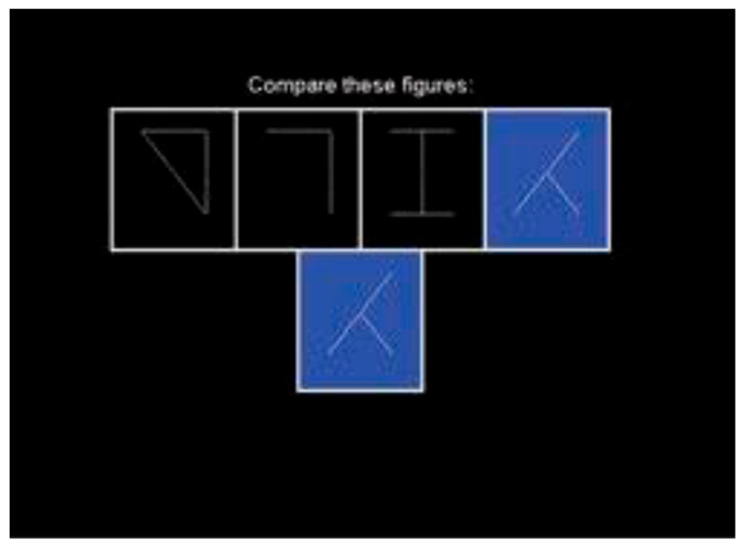
Cognitrone.

Cognitrone (Figure 2) is a test which assesses attention and concentration through comparison of figures with regard to their congruence. The participant is asked to compare an abstract figure with a model and to decide whether the two are identical. One hundred figures were presented. In this test, the respondent chooses the pace of the test because a new item/figure is presented each time the respondent enters the decision for the current item; it is not possible to correct a previous answer that had been given, and therefore the test focuses on the accuracy of performance, rather than reaction time. The main outcome from this test is total accurate responses (total correct hits + total correct rejections), with a higher score indicating better attentional performance. The test has high reliability (*r* = 0.95) [30].

**Figure 3:**
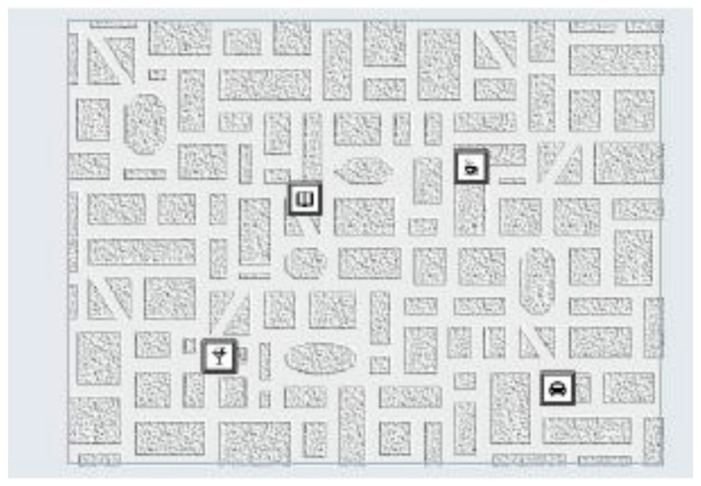
Visual memory test.

The visual memory test (Figure 3) assesses visual–spatial memory performance by measuring how respondents encode and recall visual information presented in the form of symbols on a city map. The participant is instructed to memorize positions of the individual symbols and recall them correctly afterwards. The adaptive presentation ensures that the respondents are only confronted with tasks corresponding to their performance level. The test items, created on the basis of a specific construction rationale, assesses visual memory performance. This visual memory is particularly important in the building up of so called “memory point” knowledge, which is an aspect of a person’s ability to orient themself. The presentation time, the number of presented symbols (maximum, eight) and the structure of the street map can vary in accordance with the item difficulty. The main outcome from this test is total recall, with higher recall indicating better visual spatial memory performance. The reliability of the test is *r* = 0.65 [30].

#### 2.2.2. Questionnaire Battery

Apart from the Vienna tests, the participants were also presented with a questionnaire battery in which we inquired about their demographic and lifestyle characteristics (especially physical activity). Participants were asked to provide basic demographic information, such as gender, age, education, income, marital status, height and weight (which were used to compute body mass index), and basic details of their health status. Physical activity was assessed by self-report using the Physical Activity Scale for the Elderly (PASE) [31]. The PASE records levels of physical activity in various domains, as well as types of physical activity and perceived intensity of the activity as performed in the past week. The PASE covers a broad range of leisure-time physical activity, gathers information on physical activity in work and in the household, and was designed specifically for older adults. Higher scores on the PASE indicate higher levels of physical activity.

The 12-item Short Form Health Questionnaire SF-12 [35] was used to measure self-perceived psychological and physical health. This questionnaire focuses on self-evaluation of one’s health, perceived health limits, or physical, emotional, and social aspects of one’s health (example questions: In general, would you say your health is: excellent, very good, good, fair, poor?; Do you have a lot of energy?). The SF-12 questionnaire provides subscores of self-perceived psychological health (mhs) and physical health (phs). Scores from this questionnaire represent a valid and reliable method of health evaluation and are frequently used in research on older adults [11]. As in previous work, we averaged each person’s scores on the mental and physical subtests in order to derive a single overall self-perceived-health score for each participant representing both physical and mental domains [36].

### 2.3. Statistical Analyses

Using linear regressions, we examined the relationship between physical activity, self-perceived health, and cognitive performance. Gender and age were included as covariates in all models.

We next tested whether processing speed could explain the variance between physical activity and self-perceived health using the PROCESS macro for SPSS (SPSS Statistics Armonk, NY, US). Processing speed was tested as a statistical intermediary of the relationship between physical activity and self-perceived health. Physical activity was entered as the independent variable and perceived health was entered as the dependent variable. Age and gender were again included as covariates.

## 3. Results

### 3.1. Physical Activity and Self-Perceived Health

**Figure 4:**
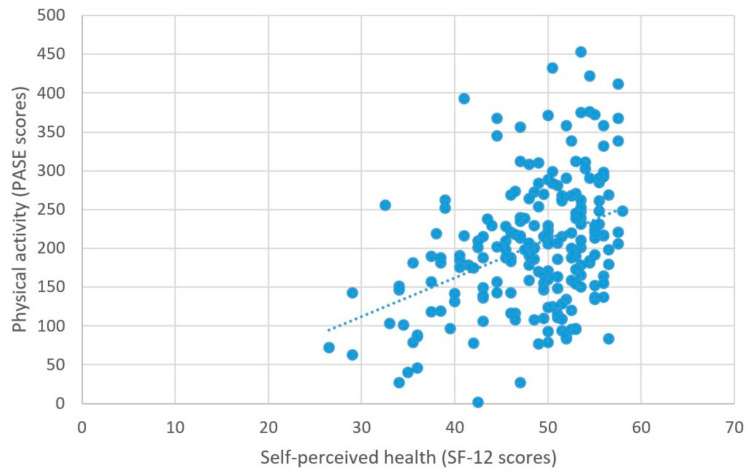
Physical activity and self-perceived health.

As predicted, better self-perceived health was associated with higher physical activity levels, β = 0.03, *t* = 5.2, *p* < 0.001 (Table 2; Figure 4).

### 3.2. Physical Activity and Cognitive Performance

**Figure 5:**
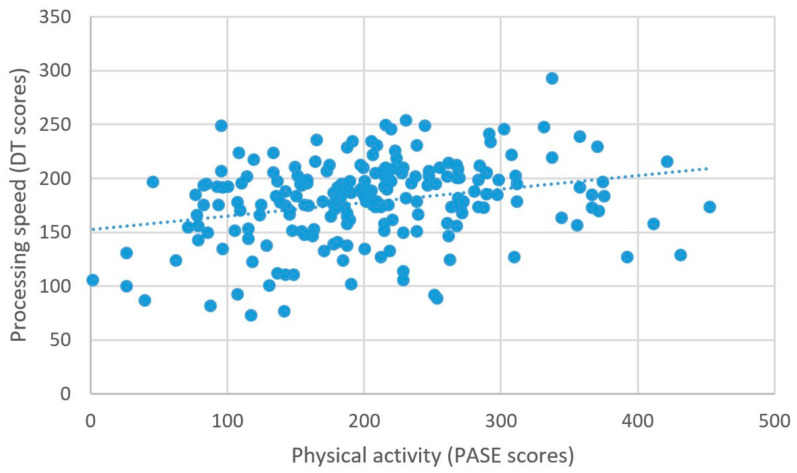
Physical activity and processing speed.

Higher self-reported physical activity was associated with higher scores on the measure of processing speed, β = 0.074, *t* = 2.77, *p* = 0.006 (Figure 5). There were no other significant relationships between physical activity and the other measures of cognitive performance; all βs < 0.01, ps > 0.08 (Table 2).

### 3.3. Self-Perceived Health and Cognitive Performance

**Figure 6:**
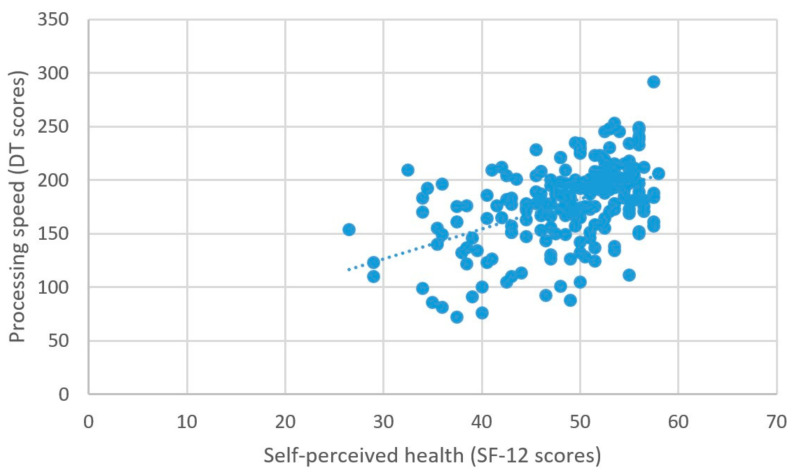
Self-perceived health and processing speed.

**Figure 7:**
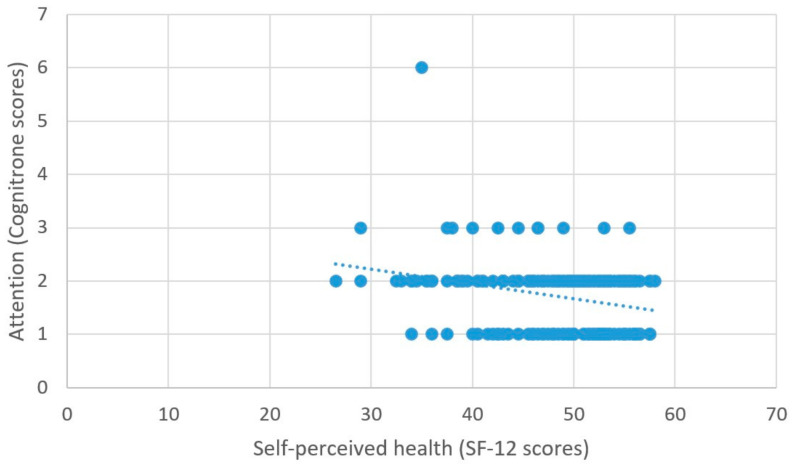
Self-perceived health and attention.

**Figure 8:**
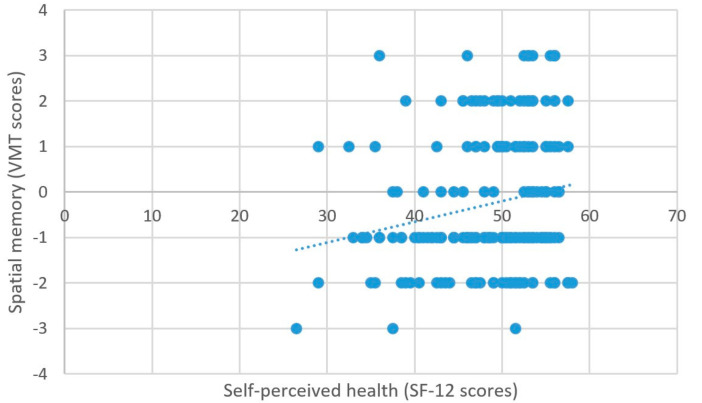
Self-perceived health and spatial memory.

As predicted, better self-perceived health was associated with higher scores on the measure of processing speed (β = 2.03, *t* = 5.99, *p* < 0.001) (Figure 6), better attentional performance (β = 0.15, *t* = 2.89, *p* = 0.001) (Figure 7), and better visual spatial memory (β = 0.06, *t* = 2.44, *p* = 0.02) (Table 3; Figure 8).

### 3.4. PROCESS Results

The measure of processing speed was the only test that was significantly associated with both physical activity and self-perceived health; therefore, it qualified as a variable that potentially accounts for the relationship between these latter two variables [37]. Thus, processing speed was entered as the intermediary variable in a simple mediation model. Physical activity and self-perceived health were entered as the independent and dependent variables, respectively.

**Figure 9:**
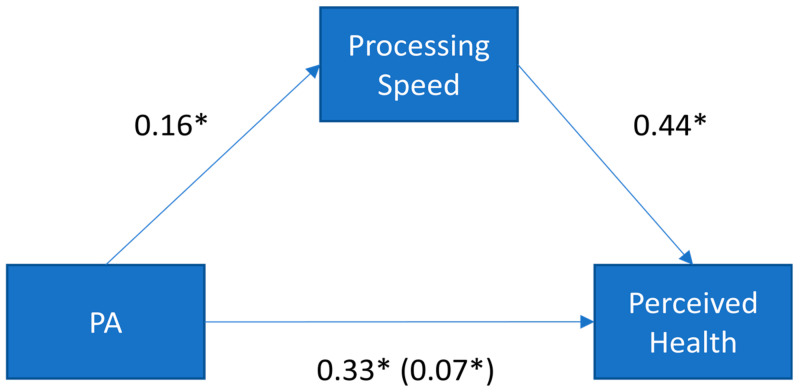
Standardized regression coefficients for the relationship between physical activity (PA) and perceived health. The standardized regression coefficients between PA and self-perceived health, controlling for processing speed, are presented in parentheses. * *p* < 0.05.

Importantly, we found that processing speed completely accounted for the relationship between physical activity and self-perceived health, as measured by the significance of the indirect effect. As Figure 9 illustrates, the standardized indirect effect was 0.07. PROCESS tests the significance of the indirect effect using bootstrapping. Specifically, unstandardized indirect effects are computed for each of 10,000 bootstrapped samples, and the 95% confidence interval is computed by determining the indirect effects at the 2.5th and 97.5th percentiles. The bootstrapped unstandardized indirect effect was 0.005, and the 95% confidence interval ranged from 0.002 to 0.009. Thus, because the confidence intervals associated with the indirect effect do not include zero, the effect was statistically significant.

Due to the cross-sectional nature of these data, we conducted a follow-up analysis to examine the possibility of reverse directionality. Specifically, we tested the alternative possibility that physical activity could account for the relationship between self-perceived health and cognitive functioning (thus, flipping the model to consider physical activity as the intermediary variable rather than the independent variable). The results of this model were not significant (indirect effect = 0.004; LLCI, ULCI = −0.003, 0.012), supporting the hypothesized placement of these variables in the original model.

## 4. Discussion

As predicted, we found that better self-perceived health was associated with higher physical activity levels. These findings are consistent with the existing literature, demonstrating a positive relationship between these variables [3,29]. Higher self-perceived health was also associated with better cognitive performance across all three cognitive domains assessed (processing speed, spatial memory, and attention). To the best of our knowledge, there is only one existing study examining the relationship between physical health (including self-perceived health and functional abilities) and cognitive performance in healthy older adults [35]. In that study, Van Hooren et al. [35] found that higher self-perceived health was associated with better cognitive performance across both processing speed and memory domains. The self-perceived health-cognition relationships we report are consistent with this earlier study and suggest that people who have better processing speed might be more aware of their physical health status. This is important because it suggests that changes in self-perceived health might be an indicator of cognitive change. However, no studies to date had examined physical activity, cognitive function, and perceived health in the same study, nor had any examined cognitive function as a potential mechanism linking physical activity to perceived health. The previously reported relationships between self-perceived health and physical activity, and self-perceived health and cognition led to our novel research question: might the relationship between physical activity and self-perceived health be accounted for by variability in cognitive function? The results of our model suggest that this may indeed be the case.

The most novel finding in the present paper was that processing speed statistically accounted for the relationship between physical activity and self-perceived health, suggesting that it might be a possible mechanism for the relationship between physical activity and self-perceived health. Importantly, the alternative possibility that physical activity accounts for the relationship between self-perceived health and cognitive functioning was not supported by the data. This pattern of results suggests that physical activity is associated with better processing speed, which, in turn, is associated with a greater perception of overall health. Future interventions that manipulate physical activity and examine both self-perceived health and cognition would provide a more causal test of these associations.

Partially confirming our hypothesis, we found that physical activity was positively related to processing speed, but not to other cognitive measures. The relationship between physical activity and cognitive function is especially robust for measures of executive function, and so the lack of relationship with executive functioning measures in our data is surprising [25]. However, this lack of a relationship might be due to the characteristics of the tests used. For example, the measures of visual memory and attention did not include a timed component (i.e., requiring processing speed), which is perhaps one explanation for why a physical activity–attention or physical activity–memory relationship was not detected. However, our finding of an association with processing speed suggests that aspects of basic cognitive speed are positively associated with participation in leisure time physical activity. Processing speed is considered a critical component of cognitive aging [28,32], and many measures of executive function and attention include a speed or timed component, the association with physical activity might suggest a possible common link by which physical activity influences other cognitive domains (e.g., executive function).

### Limitations

***Limited Directional Information Based on the Study Design.*** An important limitation of this work is the cross-sectional nature of the study. Indeed, it is impossible to infer directionality. For example, it is possible that higher self-perceived health leads to higher physical activity levels (rather than the reverse). We statistically tested for this alternative possibility in the statistical model by moving physical activity into the intermediary position rather than considering it as the independent variable. This model was not significant, lending support to the placement of the variables in the model. Nonetheless, these data are inherently correlational. Thus, while the mediation analysis we report supports our hypothesis about the links between these variables, we cannot make any conclusions about causality. In fact, some of the reported relationships could very well be bidirectional. Future research should examine these relationships in an intervention design to make more definitive causal conclusions regarding directionality.

***Self-reported physical activity.*** Due to the relatively large sample size of the present study, we chose to collect self-reported physical activity via a validated questionnaire. However, we acknowledge that other measures of physical activity, such as using objective monitoring methods, might paint a different picture of these associations and characterize physical activity levels differently. Despite the methodological choice to collect self-reported physical activity, we demonstrated relationships that are consistent with other research using both self-reported and device-measured physical activity.

***Unmeasured variables.*** We controlled for age and sex in all of our analyses because these variables are known to be associated with both physical activity and cognition, and because our sample was predominately female. However, there are a number of other, unmeasured variables that could covary with physical activity, cognition, and perceived health, and therefore could have confounded the relationships reported. For example, there are other health conditions, such as depression, anxiety, or cardiovascular conditions that may covary with the variables in our model. Indeed, these conditions may be more prevalent in older adults. Unfortunately, we did not collect a detailed medical history on this sample. Doing so would be an important avenue for future research. Another related limitation is that we did not have a comparison group of a different age range (e.g., younger adults). Thus, it is not possible to tell whether these results would generalize across lifespans.

## 5. Conclusions

Despite the limitations noted above, we can draw several broad conclusions from these results. In particular, the results of the study support the importance of physical activity as a health behavior that is linked to both faster processing speed and elevated self-perceptions of health. Based on the results from the statistical mediation model, we argue that greater levels of physical activity might be indirectly linked to self-perceptions of health via measures of processing speed.

## Figures and Tables

**Table 1:** Demographic characteristics of the sample.

Age	Mean (SD)	70.02 (6.092)
Gender	Females	75%
	Males	25%
Education	Elementary	3.4%
	High school	74.1%
	University	22.5%
Physically active according to the WHO		67%
Marital status	Married/living with partner	60.3%
	Widowed	24.5%
	Divorced/separated	12.3%
	Single	2.5%
Number of children	Mean (SD)	2.181 (0.867)
BMI	Mean (SD)	26.92 (4.372)
Health problems	(osteoarthritis, high blood pressure, higher cholesterol, diabetes II. degrees, cardiac arrhythmias, joint pain, and various postoperative conditions)	66.7%
Medication		71.6%

**Table 2:** Results of regressions examining the relationship of physical activity to self-perceived health and cognitive function. Age and sex are included as covariates.

Outcome Variable	Unstandardized Beta	Standardized Beta	*t*	*p*
Self-perceived health	0.03	0.33	5.2	<0.001
Processing speed	0.07	0.16	2.8	0.006
Attention	0.01	0.12	1.8	0.08
Visuospatial memory	0.00	−0.12	1.7	0.09

**Table 3:** Results of regressions examining the relationship of perceived health to cognitive performance. Age and sex are included as covariates.

Outcome Variable	Unstandardized Beta	Standardized Beta	*t*	*p*
Processing speed	2.03	0.34	5.99	0.006
Attention	0.15	0.20	2.89	0.005
Visuospatial memory	0.05	0.16	2.44	0.02
